# Body composition is associated with risk of toxicity-induced modifications of treatment in women with stage I–IIIB breast cancer receiving chemotherapy

**DOI:** 10.1007/s10549-018-5014-5

**Published:** 2018-10-23

**Authors:** Maaike M. G. A. van den Berg, Dieuwertje E. Kok, Liesbeth Posthuma, Lisette Kamps, Celine S. Kelfkens, Nicole Buist, Maud Geenen, Annebeth Haringhuizen, Joan B. Heijns, Rianne H. M. A. van Lieshout, Maartje Los, Dirkje W. Sommeijer, Johanna N. H. Timmer-Bonte, Anja Th. C. M. de Kruif, Hanneke W. M. van Laarhoven, Ellen Kampman, Renate M. Winkels

**Affiliations:** 1 Division of Human Nutrition and Health, Wageningen University & Research, PO Box 17, 6700 AA Wageningen, The Netherlands; 2 Amphia Ziekenhuis, Breda, The Netherlands; 3 Sint Lucas Andreas Ziekenhuis, Amsterdam, The Netherlands; 4 Ziekenhuis Gelderse Vallei, Ede, The Netherlands; 5 Máxima Medical Centre, Eindhoven, The Netherlands; 6 St Antonius Ziekenhuis, Nieuwegein, The Netherlands; 7 Flevoziekenhuis, Almere, The Netherlands; 8 Department of Oncology, Alexander Monro Ziekenhuis, Bilthoven, The Netherlands; 9 Department of Health Sciences, VU University, Amsterdam, The Netherlands; 10 Department of Oncology, Academic Medical Center, Amsterdam, The Netherlands; 11 Department of Public Health Sciences, Penn State College of Medicine, Hershey, PA USA

**Keywords:** Chemotherapy, Toxicity, Breast cancer, Body composition, Fat mass

## Abstract

**Purpose:**

Initial dose of chemotherapy is planned based on body surface area, which does not take body composition into account. We studied the association between fat mass (kg and relative to total body weight) as well as lean mass (kg and relative to total body weight) and toxicity-induced modifications of treatment in breast cancer patients receiving chemotherapy.

**Methods:**

In an observational study among 172 breast cancer patients (stage I–IIIB) in the Netherlands, we assessed body composition using dual-energy X-ray scans. Information on toxicity-induced modifications of treatment, defined as dose reductions, cycle delays, regimen switches, or premature termination of chemotherapy, was abstracted from medical records. Adjusted hazard ratios and 95% confidence intervals (95% CI) were calculated to assess associations between body composition and the risk of toxicity-induced modifications of treatment.

**Results:**

In total, 95 out of 172 (55%) patients experienced toxicity-induced modifications of treatment. Higher absolute and relative fat mass were associated with higher risk of these modifications (HR 1.14 per 5 kg; 95% CI 1.04–1.25 and HR 1.21 per 5%; 95% CI 1.05–1.38, respectively). A higher relative lean mass was associated with a lower risk of modifications (HR 0.83 per 5%; 95% CI 0.72–0.96). There was no association between absolute lean mass and risk of toxicity-induced modifications of treatment.

**Conclusions:**

A higher absolute and a higher relative fat mass was associated with an increased risk of toxicity-induced modifications of treatment. Absolute lean mass was not associated with risk of these treatment modifications, while higher relative lean mass associated with lower risk of modifications. These data suggest that total fat mass importantly determines the risk of toxicities during chemotherapy in breast cancer patients.

**Electronic supplementary material:**

The online version of this article (10.1007/s10549-018-5014-5) contains supplementary material, which is available to authorized users.

## Introduction

Breast cancer patients are often treated with chemotherapy, which usually consist of a combination of anthracyclines (e.g., doxorubicin, epirubicin) and taxanes (e.g., paclitaxel, docetaxel) with or without targeted therapy [1, 2]. Severe side effects during chemotherapy can lead to a dose reduction, cycle delay, or premature termination of treatment. These toxicity-induced modifications of treatment may eventually lead to a reduced dose intensity, and worse outcome [3].

In clinical practice, the administered dose of the chemotherapy is based on the body surface area. Body surface area is usually calculated using the Mosteller formula based on height and weight [4]. However, it does not distinguish lean mass from fat mass or other characteristics of body composition. It has been suggested that body composition may be more important than body surface area for calculating the administered dose of chemotherapy, since previous studies in other cancer types showed that patients with low lean mass have a higher risk of toxicities as a result of treatment [5–9].

In patients with metastatic breast cancer, sarcopenia as well as low muscle mass or low lean mass were associated with increased risk of toxicities during chemotherapy [10–12]. Body composition of metastatic breast cancer patients generally differs from early stage breast cancer patients, because of disease-related sarcopenia and/or cachexia. Therefore, findings in metastatic breast cancer may not be generalizable to early stage breast cancer [10, 12, 13].

So far, only two studies on body composition in association with treatment-related toxicities in early breast cancer patients were published. Both studies suggested that a lower lean mass was associated with an increased risk of toxicities [14, 15].

The goal of this paper was to focus not only on lean mass, but also to study associations between fat mass and risk of toxicities. Thus, we aimed to assess the association between fat mass, lean mass, and the risk of toxicity-induced modifications of treatment [16] in women with stage I-IIIB breast cancer receiving (neo)adjuvant chemotherapy.

## Methods

### Participants

This study is part of the COBRA-study, an observational multi-centre study among breast cancer patients receiving (neo)adjuvant chemotherapy [17]. Eligible patients were recruited by the medical staff from 11 academic and peripheral hospitals in the Netherlands prior to commencement of chemotherapy. Women were eligible if they were newly diagnosed with operable stage I–IIIB breast cancer, and scheduled to receive (neo)adjuvant chemotherapy. Participants needed to be at least 18 years old and be able to communicate in Dutch. Exclusion criteria were history of cancer, previous treatment with chemotherapy, (intended) pregnancy during the study period, dementia, or other mental conditions that made it impossible to comply with the study procedures.

For the current analyses, data were available for 176 breast cancer patients recruited between May 2013 and September 2016. Four patients had to be excluded, because they had no dual-energy X-ray absorption (DEXA)-scan available. In total, we considered 172 patients for the analyses for this study.

The study protocol was approved by the Medical Ethical Committee of Wageningen University & Research, the Netherlands. All participants provided written informed consent.

### Data collection

#### Body composition

Body composition was assessed using a dual energy X-ray absorptiometry (DEXA) scan. Participants were measured in the hospitals by trained technicians using a total body scan protocol prior to start of chemotherapy (*n* = 86) or during the first cycle of chemotherapy (*n* = 86). Based on the total body DEXA-scan body weight (kg), fat mass (kg and relative to total body weight), and lean mass (kg and relative to total body weight) were assessed. Body composition data were included in the models as continuous variables. In addition, we categorized patients into tertiles of lean mass, and tertiles of fat mass (Supplementary Tables 2 and 3). Using this categorization, we defined the lowest tertile of lean mass as “low lean mass,” and the upper two tertiles as “normal lean mass;” in addition, the highest tertile of fat mass was defined as “high fat mass” and the lower two tertiles as “normal fat mass.” Based on this categorization, we created four groups: (1) patients with a normal lean mass and normal fat mass, (2) patients with a normal lean mass and high fat mass, (3) patients with low lean mass and normal fat mass, or (4) patients with low lean mass and high fat mass.

### Toxicity-induced modifications of treatment

Information regarding chemotherapy and toxicity was abstracted from medical records using a standardized form. Treatment information included detailed information on type and dose of chemotherapy, number of cycles planned, and start dates of each cycle. Furthermore, information on actual administered dose, toxicities, and reasons for treatment modifications were collected per cycle. Toxicity-induced modifications of treatment were defined as dose reductions, cycle delays, or premature termination of chemotherapy [16]. Also, if a planned cytotoxic regime was changed to another regime because of toxicities, this was reported as a treatment modification. If the reason for a dose reduction or cycle delay was unknown (*n* = 4), we included them as toxicity-induced modifications of treatment. Logistical or other non-medical reasons for cycle delays were not classified as toxicity-induced modification of treatment.

### Patient and clinical characteristics

Information about tumour stage at diagnosis and timing of chemotherapy (adjuvant versus neo-adjuvant) was collected from medical records. Information regarding age at cancer diagnosis and height was collected using a general questionnaire. Based on body weight of the DEXA-scan and self-reported height, BMI at diagnosis was calculated. Chemotherapy regimens were categorized as combined or sequential regimes (Supplementary Table 1); combined regimes included schemes with different components administered together during all cycles, and included TAC, FEC, DOC-CYCLO, CDT(P) PT, and CTP. Sequential regimes included schemes with different components that were administered in different cycles and included AC/P(T), FEC/DOC, and AC/DOC/(T).

### Data analysis

Population characteristics are presented as median with interquartile range (IQR) or number with percentage for the total study population, and participants experiencing a toxicity-induced modification of treatment (yes vs. no) separately.

Hazard ratios (HRs) and 95% CI were calculated to assess the association between body composition and time to treatment modification using a Cox proportional hazard regression model; time was expressed as the number of completed cycles of chemotherapy until there was treatment modification. For example, if a patient had 6 planned cycles of chemotherapy and did not experience any toxicity-induced modification of treatment, time was censored at 6 cycles. The proportional hazard assumption was checked and was not violated, as evaluated by the goodness-of-fit test using Schoenfeld residuals (*p* > 0.05).

For all analyses, we constructed a separate model for each body composition parameter, i.e., BMI (kg/m^2^), fat mass per 5%, fat mass per 5 kg, lean mass per 5%, and lean mass per 5 kg. In addition, we assessed HRs for the following four groups: (1) patients with a normal lean mass and normal fat mass versus, (2) patients with a normal lean mass and high fat mass, (3) patients with low lean mass and normal fat mass, or (4) low lean mass and high fat mass. Stratified analyses were conducted for patients receiving a sequential regime versus a combined regime, and for patients receiving adjuvant chemotherapy versus neo-adjuvant chemotherapy. A sensitivity analysis was conducted for toxicity-induced treatment modifications occurring within the first 6 cycles of chemotherapy. This was done to account for the fact that patients with a higher number of cycles planned have higher odds of experiencing toxicities as they go through more cycles. In this sensitivity analysis, only toxicity-induced treatment modifications occurring within the first 6 cycles were considered.

Analyses were adjusted for age, since older women have an increased risk of experiencing toxicities and age is associated with specific body composition characteristics [18]. Based on literature, BSA was considered as potential covariate, but not included in the analyses since BSA was strongly related with the body composition parameters (multicollinearity). All analyses were performed using SAS version 9.4 (SAS Institute, Inc., Cary, NC, USA).

## Results

The median age of the 172 women included was 51.8 years (Table 1). More than half of the patients were overweight or obese at diagnosis. Most patients had a stage II tumour, received adjuvant chemotherapy with a sequential regime consisting of 6 or less planned cycles. Table 1 shows that women experiencing a toxicity-induced modification of treatment were more often treated with a sequential regime of adjuvant chemotherapy compared to the women not experiencing a treatment modification. In addition, women experiencing a toxicity-induced modification of treatment had a higher body weight, were more often overweight or obese, had a higher fat mass and lower percentage of lean mass compared to the women not experiencing a modification.

**Table 1:** Demographic, clinical, and body composition characteristics of breast cancer patients treated with chemotherapy

	Total (*n* = 172)	Toxicity-induced modification of treatment: yes (*n* = 95)	Toxicity-induced modification of treatment: no (*n* = 77)	*P* value*
Demographics				
Age, years (median, IQR)	51.8 (46.8; 59.1)	52.1 (47.4; 60.8)	51.5 (46.4; 54.6)	0.16
Medical profile				
Stage (*n*, %)				0.82
I	44 (25.6)	26 (27.4)	18 (23.4)	
II	105 (61.1)	57 (60.0)	48 (62.3)	
III	23 (13.4)	12 (12.6)	11 (14.3)	
Chemotherapy (*n*, %)				0.68
Adjuvant	111 (64.5)	67 (70.5)	44 (57.1)	
Neo-adjuvant	61 (35.5)	28 (29.5)	33 (42.9)	
Type of chemotherapy (*n*, %)				0.03
Combined regime	78 (45.3)	36 (37.9)	42 (54.6)	
Sequential regime	94 (54.7)	59 (62.1)	35 (45.5)	
Number of cycles chemotherapy (*n*, %)				< 0.01
6 or less	119 (69.2)	57 (60.0)	62 (80.5)	
More than 6	53 (30.8)	38 (40.0)	15 (19.5)	
Anthropometry and body composition				
Body weight, kg (median, IQR)	70.5 (63.9; 81.7)	74.1 (64.4; 84.6)	68.2 (63.1; 76.1)	0.04
Height, cm (median, IQR)	168 (164; 173)	168 (162; 173)	168 (164; 173)	0.30
Body surface area (BSA), (median, IQR)	1.8 (1.7; 2.0)	1.8 (1.7; 2.0)	1.8 (1.7; 1.9)	0.08
Body mass index (BMI) kg/m^2^ (median, IQR)	25.5 (22.5; 29.1)	26.5 (23.9; 29.8)	24.5 (21.7; 27.2)	< 0.01
Fat mass, percentage (median, IQR)	36.7 (31.4; 42.2)	38.6 (33.7; 44.8)	35.0 (29.7; 39.9)	< 0.01
Fat mass, kg (median, IQR)	26.0 (20.2; 34.2)	27.6 (20.8; 36.3)	23.1 (18.4; 31.1)	0.01
Lean mass, percentage (median, IQR)	60.2 (33.2; 65.1)	58.5 (53.1; 63.5)	61.8 (57.2; 66.7)	< 0.01
Lean mass, kg (median, IQR)	43.1 (29.4; 46.8)	43.1 (39.5; 47.5)	42.8 (39.3; 46.6)	0.76
Appendicular skeletal mass, kg (median, IQR)	18.2 (16.8; 20.2)	18.2 (16.6; 20.2)	18.2 (16.9; 20.1)	0.83
Skeletal muscle index, kg/m^2^ (median, IQR)	6.5 (6.0; 7.2)	6.5 (6.0; 7.3)	6.4 (6.; 7.1)	0.56

Data are given for the total group and stratified by “Toxicity-induced modification of treatment” Yes or No

*Mann-Witney *U* for continuous variables, *χ*^2^ for categorical variables

Table 2 specifies how often the specific types of treatment modifications occurred. During chemotherapy, more than half of the patients experienced an adjustment in relative dose intensity (95 out of 172 patients, 55%). Of these 95 patients, 14% (*n* = 13) stopped prematurely, 53% (*n* = 50) had a dose reduction, and 34% (*n* = 32) had a cycle delay as their first toxicity-induced modification of treatment. In total, 48% (*n* = 57) of the women receiving 6 or less planned cycles experienced a toxicity-induced modification versus 72% (*n* = 38) of the women receiving more than 6 planned cycles.

**Table 2:** Distribution of the three types of treatment modifications as experienced by breast cancer patients in the COBRA study among patients who had no more than 6 planned chemotherapy cycles versus more than 6 cycles

Toxicity	All patients (*n* = 172)	6 planned cycles or less (*n* = 119)	More than 6 planned cycles (*n* = 53)
Any modification, *n* (%)	*95* (*55.2*)	*57* (*47.9*)	*38* (*71.7*)
Cycle delay	32 (33.7)	17 (29.8)	15 (39.5)
Dose reduction	50 (52.6)	36 (63.2)	14 (36.8)
Premature termination	13 (13.7)	4 (7.0)	9 (23.7)

A higher BMI was associated with a higher risk of toxicity-induced modifications of treatment (HR 1.06 per kg/m^2^ (95% CI 1.02–1.11). Higher absolute fat mass was associated with higher risk of modifications [HR per 5 kg fat mass: 1.14 (95%CI 1.04–1.25)]. Higher relative fat mass was also associated with a higher risk of modifications [HR per 5% increase in fat mass: 1.21 (95% CI 1.05–1.38)], see Table 3. A higher percentage of relative lean mass was associated with a lower risk of toxicity-induced modification of treatment [HR per 5 percent increase in lean mass: 0.83 (95% CI 0.72–0.96)]. Absolute lean mass in kg was not associated with treatment modifications (Table 3).

**Table 3:** Association between body composition parameters and risk of toxicity-induced modifications of treatment in early stage breast cancer patients

Variable	Total/cases	HR	95% CI
All patients			
BMI per kg/m^2^	172/95	**1.06**	**1.02; 1.11**
Fat mass per 5%	172/95	**1.21**	**1.05; 1.38**
Fat mass per 5 kg	172/95	**1.14**	**1.04; 1.25**
Lean mass per 5%	172/95	**0.83**	**0.72; 0.96**
Lean mass per 5 kg	172/95	1.07	0.92; 1.27
Appendicular skeletal mass per kg	172/95	1.01	0.95; 1.08
Skeletal muscle index per kg/m^2^	172/95	1.07	0.87; 1.32
Sensitivity analysis: modifications in the first 6 cycles			
BMI per kg/m^2^	172/73	**1.06**	**1.01; 1.11**
Fat mass per 5%	172/73	**1.18**	**1.01; 1.37**
Fat mass per 5 kg	172/73	**1.13**	**1.02; 1.25**
Lean mass per 5%	172/73	**0.85**	**0.72; 1.00**
Lean mass per 5 kg	172/73	1.08	0.91; 1.30
Appendicular skeletal mass per kg	172/73	1.02	0.95; 1.09
Skeletal muscle index per kg/m^2^	172/73	1.10	0.88; 1.38

HRs were adjusted for age

Bold values are statistically significant

A total of 54 patients had a low lean mass in combination with high fat mass (Table 4). Having a low lean mass in combination with high fat mass was associated with a higher risk of toxicity-induced modifications of treatment versus having a normal lean mass in combination with normal fat mass (HR 1.33, 95% CI 1.01–1.75).

**Table 4:** Categorization of patients into body composition groups and association of body composition groups with risk of toxicity-induced modifications of treatment in early stage breast cancer patients

Group	Total	Toxicity-induced modification of treatment: yes	Toxicity-induced modification of treatment: no	HR
Normal lean and normal fat	111	55	56	Ref
Normal lean and high fat*	4	2	2	–
Low lean and normal fat*	3	1	2	–
Low lean and high fat	54	37	17	1.33 (1.01, 1.75)

Categorization is based on tertiles with “normal lean mass” defined as the upper two tertiles of lean mass, “normal fat mass” as the lowest two tertiles of fat mass, “low lean mass” as the lowest tertile of lean mass and “high fat mass” as the upper tertile of fat mass

*Not included in Cox Proportional hazard analysis, because of low numbers. HR adjusted for age

**Fig. 1:**
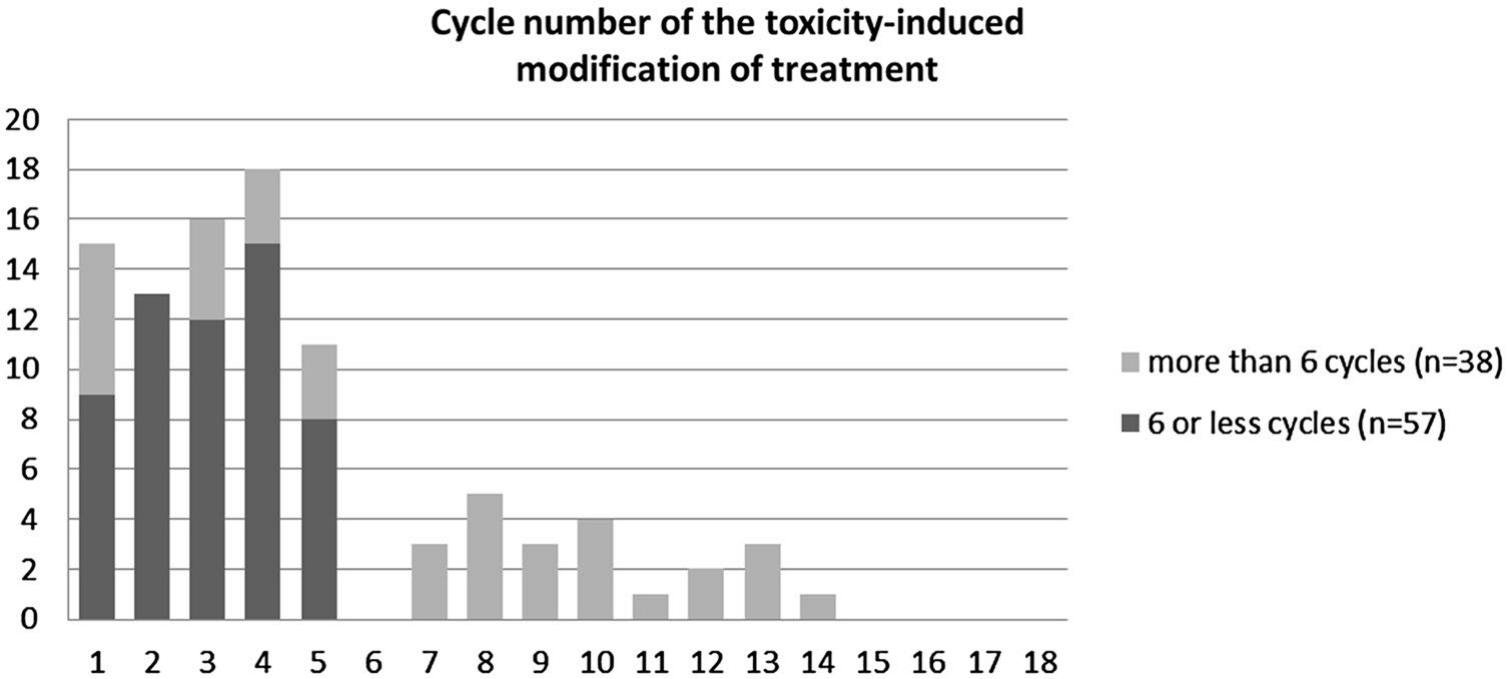
Cycle number in which the first toxicity-induced modification of treatment occurred, stratified for patients receiving a chemotherapy regime consisting of 6 or less planned cycles and patients receiving a chemotherapy regime consisting of more than 6 planned cycles

Figure 1 shows the time in cycle numbers until the occurrence of the first toxicity-induced modification of treatment. In total, 73 of the 95 women (77%) experienced their first modification within the first 6 cycles of chemotherapy. Sensitivity analyses including only modifications occurring within the first 6 cycles showed similar results compared to the analysis in which we included modifications in all cycles (Table 3).

Stratified results did not suggest that the associations between body composition and toxicity-induced modifications of treatment were different for combined versus sequential regimes, nor for neo-adjuvant versus adjuvant chemotherapy (data not shown). We had insufficient power to stratify by specific chemotherapy regimens, but none of the regimens appeared more related to toxicity profiles than others (Supplementary Table 1).

## Discussion

This study showed that a higher absolute or relative fat mass and a lower percentage of lean mass were associated with an increased risk of toxicity-induced modifications of treatment in stage I-IIIB breast cancer patients treated with chemotherapy, while absolute lean mass in kg was not associated with these modifications. Our results suggest that fat mass strongly determines the risk of treatment modifications during chemotherapy in breast cancer patients.

Two earlier studies [14, 15] stressed the importance of total lean mass in the association with chemotherapy-induced toxicities. However, those studies did not assess chemotherapy-induced toxicities in association with relative lean mass or total fat mass (kg or percentage). The first study (*n* = 151) used CT-scans to assess body composition and concluded that lower total lean mass and skeletal muscle gauge—a composite endpoint of muscle mass and muscle radio density—were associated with increased risk of treatment-related grade 3–4 toxicities in patients receiving doxorubicin-cyclophosphamide (AC)-taxane-based cytotoxic regimens [14]. The second study (*n* = 24) concluded that a lower total lean mass was associated with higher incidence of dose-limiting toxicities during the first cycle of FEC100 [15], again with CT-scan as measurement of body composition. Both studies extrapolated total lean mass from skeletal muscle cross-sectional area of a CT-scan at the level of the third vertebrae, but did not report results of toxicity associations with percentage of total lean mass or fat mass. Moreover, the study populations of both studies differed from our population, making it challenging to compare the results. For example, in the study of Prado 22 out of 24 patients experienced an adjustment in relative dose during the first cycle of chemotherapy [15], which is considerably more than our study where 15 out of 172 patients experienced a toxicity-induced modification of treatment after the first cycle. This suggests that the selection process of participants eligible for their study led to a group of patients at high risk of toxicities which may impact the generalizability of those findings. In the study by Shachar et al, the average BMI was 2–3 kg/m^2^ higher than in our study, while lean mass was slightly lower [14]. Thus, it is plausible that fat mass was higher in that study, and that patients with lower lean, and/or higher fat mass experienced the highest risk of toxicities, in line with the results of our current study. Yet, baseline differences in body composition between studies plus different outcome measures to assess toxicities obstruct direct comparison between studies. We could not differentiate between visceral and subcutaneous abdominal adiposity with our DEXA data. New algorithms are emerging that enable this distinction, which opens up avenues for further study [19].

Possible mechanisms for the observed association between body composition and toxicity-induced modifications of treatment are unclear, but could be either biological or clinical. Depending on the type of cytotoxic agent, drugs may be more hydrophilic or hydrophobic which will affect the clearance and volume of distribution of the drugs. For hydrophilic drugs, it has been hypothesized that patients with a relatively lower lean mass may be overdosed when using body surface area to calculate dosage, and may present with higher rates of toxicity-induced modifications of treatment. In our study, stratified results based on type of chemotherapy did not suggest that associations between body composition and toxicity-induced modifications of treatment were different for combined versus sequential regimes, but we did not have sufficient statistical power for further stratifications.

A more clinical, although speculative, explanation for a higher risk of toxicities in patients with low lean mass could be that clinicians treat patient with a lower percentage of lean mass differently than patients with a higher percentage lean mass, although lean mass is seldom formally assessed in clinical practice. Nevertheless, patients with a low lean mass may be frailer, and may generally experience other comorbidities, which could prompt the medical oncologist to adapt the chemotherapy protocol earlier than patients with a better physical condition.

In conclusion, a higher BMI and a higher fat mass (kg and percentage) are associated with an increased risk of toxicity-induced modifications of treatment, while absolute lean mass was not associated with risk of these modifications. This suggests that total fat mass strongly determines the risk of toxicity-induced modifications of treatment during chemotherapy in breast cancer patients. These findings highlight the importance of fat mass in relation to toxicities and provide unique leads for further clinical studies focussing on implementation of body composition measures during planning of chemotherapy.

## Electronic supplementary material

Below is the link to the electronic supplementary material.

Supplementary material 1 (DOCX 23 KB)
