# Supplementary material for: Body composition is associated with risk of toxicity-induced modifications of treatment in women with stage I–IIIB breast cancer receiving chemotherapy
Source: Breast Cancer Res Treat. 2018 Oct 23;173(2):475–81. doi: 10.1007/s10549-018-5014-5 (PMC6394786; doi:10.1007/s10549-018-5014-5)
Supplement: Supplementary file 1 — Supplementary material 1 (DOCX 23 KB) [file 10549_2018_5014_MOESM1_ESM.docx]

**Supplementary table 1: Chemotherapy regimens of breast cancer patients participating in a study on body composition and toxicity-induced modifications of treatment**

**Van den Berg et al, 2018**

|  | Chemotherapy | Scheme | Number of patients on this regimen & number of patients with a toxicity-induced modification of treatment |
| --- | --- | --- | --- |
| Combined regimens | TAC | 6 times docetaxel, doxorubicin and cyclophosphamide every 3 weeks | 63 / 26 |
|  | FEC | 6 times fluorouracil, epirubicin and cyclophosphamide every 3 weeks | 2 / 1 |
|  | DOC-CYCLO | 6 times docetaxel and cyclophosphamide every 3 weeks | 3 / 3 |
|  | CDT(P) | 6 times carboplatin, docetaxel and trastuzumab with or without pertuzumab every 3 weeks | 1 / 0 |
|  | PT | 12 times paclitaxel and trastuzumab every week | 5 / 3 |
|  | CTP | 18 times carboplatin, paclitaxel and trastuzumab, every week | 3 / 2 |
|  | CARBO/DOC/T | 6 times carboplatin, docetaxel and trastuzumab, every 3 weeks | 1/1 |
| Sequential regimes | AC/P(T) | 4 times doxorubicin and cyclophosphamide every 3 weeks followed by 12 times paclitaxel with or without trastuzumab every week | 35 / 26 |
|  | FEC/DOC | times fluorouracil, epirubicin and cyclophosphamide every 3 weeks, followed by 3 times docetaxel every 3 weeks | 50 / 27 |
|  | AC/DOC/(T) | 4 times doxorubicin and cyclophosphamide every 3 weeks followed by 4 times docetaxel with or without trastuzumab every 3 weeks | 9 / 6 |

**Supplementary table 2: Demographic, clinical and body composition characteristics of breast cancer patients treated with chemotherapy. Data are given stratified by tertiles of fat mass**

|  | **Tertile 1 fat**  **(n=57)** | **Tertile 2 fat**  **(n=57)** | **Tertile 3 fat**  **(n=58)** | **P-value** |
| --- | --- | --- | --- | --- |
| **Demographics**  Age, years (median, IQR) | 49.2 (41.3 ; 53.5) | 52.5 (49.1 ; 55.5) | 53.1 (49.0 ; 61.5) | <0.01 |
| **Medical profile** |  |  |  |  |
| Stage (n, %)  I  II  III | 18 (31.6)  33 (57.9)  6 (10.5) | 12 (21.1)  36 (63.2)  9 (15.8) | 14 (24.1)  36 (62.1)  8 (13.8) | 0.73 |
| Chemotherapy (n, %)  Adjuvant  Neo-adjuvant | 37 (64.9)  20 (35.1) | 35 (61.4)  22 (38.6) | 39 (67.2)  19 (32.8) | 0.81 |
| Type of chemotherapy (n, %)  Combined regime  Sequential regime | 28 (49.1)  29 (50.9) | 29 (50.9)  28 (49.1) | 21 (36.2)  37 (63.8) | 0.22 |
| Number of cycles chemotherapy (n, %)  6 or less  More than 6 | 39 (68.4)  18 (31.6) | 42 (73.7)  15 (26.3) | 38 (65.5)  20 (34.5) | 0.63 |
| **Anthropometry and body composition** |  |  |  |  |
| Body weight, kg (median, IQR) | 63.1 (57.9 ; 66.7) | 70.5 (64.4 ; 79.6) | 81.8 (74.9 ; 93.6) | <0.01 |
| Height, cm (median, IQR) | 170 (165 ; 174) | 167 (163 ; 172) | 167 (164 : 173) | 0.12 |
| Body surface area (BSA), (median, IQR) | 1.7 (1.6 ; 1.8) | 1.8 (1.7 ; 1.9) | 2.0 (1.8 ; 2.1) | <0.01 |
| Body Mass Index (BMI) kg/m^2^ (median, IQR) | 21.9 (20.6 ; 23.8) | 25.7 (23.7 ; 28.1) | 29.8 (26.6 ; 32.8) | <0.01 |
| Fat mass, percentage (median, IQR) | 29.3 (26.7 ; 31.2) | 36.7 (35.1 ; 38.9) | 45.1 (42.1 ; 48.2) | <0.01 |
| Fat mass, kg (median, IQR) | 18.3 (15.5 ; 20.4) | 25.8 (23.0 ; 29.8) | 36.0 (31.6 ; 43.3) | <0.01 |
| Lean mass, percentage (median, IQR) | 66.6 (65.2 ; 69.5) | 60.2 (58.3 ; 61.8) | 52.7 (49.7 ; 55.2) | <0.01 |
| Lean mass, kg (median, IQR) | 42.2 (40.1 ; 45.7) | 43.6 (39.3 ; 47.1) | 43.4 (39.0 ; 48.8) | 0.57 |
| Appendicular skeletal mass, kg (median, IQR) | 18.1 (17.0 ; 20.1) | 18.2 (16.5 ; 20.6) | 18.5 (16.9 ; 20.2) | 0.85 |
| Skeletal muscle index, kg/m^2^ (median, IQR) | 6.2 (5.9 ; 6.8) | 6.6 (6.3 ; 7.4) | 6.5 (6.0 ; 7.3) | 0.07 |

**Supplementary table 3: Demographic, clinical and body composition characteristics of breast cancer patients treated with chemotherapy. Data are given stratified by tertiles of lean mass**

|  | **Tertile 1**  **Lean**  **(n=57)** | **Tertile 2**  **lean**  **(n=58)** | **Tertile 3**  **lean**  **(n=57)** | **P-value** |
| --- | --- | --- | --- | --- |
| **Demographics**  Age, years (median, IQR) | 53.0 (46.5 ; 59.5) | 52.0 (48.5 ; 55.5) | 48.0 (42.0 ; 54.0) | <0.01 |
| **Medical profile** |  |  |  |  |
| Stage (n, %)  I  II  III | 12 (21.1)  37 (64.9)  8 (14.0) | 14 (24.1)  35 (60.3)  9 (15.5) | 18 (31.6)  33 (57.9)  6 (10.5) | 0.72 |
| Chemotherapy (n, %)  Adjuvant  Neo-adjuvant | 37 (64.9)  20 (35.1) | 38 (65.5)  20 (34.5) | 36 (63.2)  21 (36.8) | 0.96 |
| Type of chemotherapy (n, %)  Combined regime  Sequential regime | 24 (42.1)  33 (57.9) | 27 (46.6)  31 (53.4) | 27 (47.4)  30 (52.6) | 0.83 |
| Number of cycles chemotherapy (n, %)  6 or less  More than 6 | 38 (66.7)  19 (33.3) | 42 (72.4)  16 (27.6) | 39 (68.4)  18 (31.6) | 0.79 |
| **Anthropometry and body composition** |  |  |  |  |
| Body weight, kg (median, IQR) | 81.9 (71.8 ; 92.0) | 72.2( 64.8 ; 79.7) | 63.7 (59.5 ; 68.0) | <0.01 |
| Height, cm (median, IQR) | 167 (162 ; 172) | 167 (162 ; 172 | 170 (165 ; 174) | 0.19 |
| Body surface area (BSA), (median, IQR) | 2.0 (1.8 ; 2.1) | 1.8 (1.7 ; 1.9) | 1.7 (1.7 ; 1.8) | <0.01 |
| Body Mass Index (BMI) kg/m^2^ (median, IQR) | 29.8 (26.6 ; 32.9) | 25.6 (23.1 ; 28.2) | 22.0 (20.3 ; 23.7) | <0.01 |
| Fat mass, percentage (median, IQR) | 45.2 (42.2 ; 48.2) | 36.7 (34.8 ; 38.7) | 29.3 (26.9 ; 31.7) | <0.01 |
| Fat mass, kg (median, IQR) | 36.3 (30.2 ; 42.4) | 26.1 (22.1 ; 30.1) | 18.4 (15.9 ; 20.9) | <0.01 |
| Lean mass, percentage (median, IQR) | 52.7 (50.0 ; 55.4) | 60.2 (58.3 ; 62.0) | 66.8 (64.5 ; 69.0) | <0.01 |
| Lean mass, kg (median, IQR) | 43.2 (38.0 ; 48.4) | 43.7 (39.8 ; 47.7) | 42.2 (39.4 ; 45.0) | 0.68 |
| Appendicular skeletal mass, kg (median, IQR) | 18.4 (16.5 ; 20.3) | 18.2 (16.2 ; 20.2) | 18.1 (16.7 ; 19.6) | 0.75 |
| Skeletal muscle index, kg/m^2^ (median, IQR) | 6.4 (5.8 ; 7.1) | 6.6 (6.1 ; 7.2) | 6.2 (5.6 ; 6.7) | 0.08 |
